# Effects of Different Sources and Dietary Inclusion Levels of Astaxanthin on Growth Performance, Skin Pigmentation, and Physiological Parameters of Red Sea Bream (*Pagrus major*) Juveniles

**DOI:** 10.3390/ani16030499

**Published:** 2026-02-05

**Authors:** Arkadios Dimitroglou, Stephanie Carvajal Acevedo, Konstantina Evangelia Gleni, Athanasios Samaras, Dimitrios Barkas, Leonidas Papaharisis, Michael Pavlidis

**Affiliations:** 1 Laboratory of Applied Hydrobiology, Department of Animal Sciences, Agricultural University of Athens, Iera Odos 75, 118 55 Athens, Greece; 2 Department of Biology, University of Crete, Voutes University Campus, 700 13 Heraklion, Greece; scarcaja@uni-koeln.de (S.C.A.); biop1103@edu.biology.uoc.gr (K.E.G.); a.samaras@uoc.gr (A.S.); pavlidis@uoc.gr (M.P.); 3 Department of Research & Development, Avramar Aquaculture SA, 341 00 Chalkida, Greece; d.barkas@avramar.eu (D.B.); lpapaharisis@gmail.com (L.P.)

**Keywords:** red seabream, *Pagrus*, astaxanthin, *Haematococcus pluvialis*, *Phaffia rhodozyma*, pigmentation, skin coloration, blood parameters

## Abstract

Red seabream (*Pagrus major*) reared under intensive rearing conditions faces hypermelanosis and dyspigmentation, resulting in darker and less pink/red skin color, losing the natural appearance of the species. This has a great negative impact on consumers’ acceptance and market appeal. Two experiments were performed to test the effect of the source, level, and feeding duration of astaxanthin supplementation in the feed on red seabream skin color reversing the depigmentation problem. Firstly, three different sources of astaxanthin (artificially synthesized, algal-extracted, and yeast-extracted astaxanthin) at the same inclusion level (100 mg kg^−1^ feed) were tested. Then, the best performing source was tested using five inclusion levels (0, 20, 60, 80, and 100 mg kg^−1^) in the feed for 60 days. At the same time, growth performance, blood biochemical parameters, antioxidant capacity and cortisol levels were also assessed. The results showed that red seabream appearance can be significantly improved with the inclusion of astaxanthin from the algae *Haematococcus pluvialis* in the feed even at lower levels compared to the artificially synthesized astaxanthin for a 60-day period. Fish growth performance and blood parameters were not affected by any of the experimental treatments.

## Simple Summary

The external coloration of red seabream plays an important role in consumers’ acceptance and market appeal. Three different sources of astaxanthin, i.e., artificially synthesized, algal-extracted, and yeast-extracted astaxanthin, were included in red seabream feeds. Astaxanthin from *Haematococcus pluvialis* algae led to optimum skin color compared to the artificially synthesized and yeast-extracted astaxanthin. Within 60 days of feeding, the use of 60 mg kg^−1^ of algal astaxanthin had similar results in coloration as higher levels of artificial astaxanthin, up to 100 mg kg^−1^. This result was also accompanied by lower cortisol levels in blood.

## 1. Introduction

Consumers’ high appreciation and the high price of *Pagrus* sp., such as *Pagrus pagrus* (red porgy) [1,2], *Pagrus major* (red seabream) [3], and *Pagrus auratus* (silver seabream or Australian snapper) [4,5], have led to the introduction of these species in the aquaculture industry. These three species are considered to have significant value in aquaculture and fisheries worldwide, with a high market value [6]. Red seabream and silver seabream are important cultured species in the Asia-Pacific and Indo-Pacific region, respectively [7,8]. In 2022, aquaculture production reported a total production of 67,800 tonnes for *Pagrus auratus* mainly in Japan, a total production of 8078 tonnes for *Pagrus major* mainly in Korea, and more than 4821 tonnes for *Pagrus pagrus* mainly in Greece and Turkey [9]. Red seabream is considered a new species in Mediterranean aquaculture with a high projection that has attracted attention due to its reddish appearance, fillet quality, and great adaptability to existing farming techniques [10]. Under intensive rearing conditions, *Pagrus* sp. growth performance is satisfactory, but the main bottleneck is the external coloration of the fish [7,11]. Specifically, farmed fish are darker and less red compared to their wild conspecifics, which means that the problem is due to reduced lightness and increased redness [2,4,5]. It has been concluded that the red color of the red Sparidae is due to the accumulation of carotenoids such as astaxanthin in the skin [12]. On the other hand, no teleost is capable of endogenously synthesizing carotenoids, only modifying and storing them in several tissues of their body [13]. Hence, carotenoids must be obtained from their prey in the case of wild fish or from their feed supplemented with the appropriate quantities in the case of farmed fish [14].

Astaxanthin belongs to the xanthophyll carotenoid’s family, with a carbon skeleton of C-40 and 13 conjugated double bonds. Due to its formula, there are structural differences in the geometric isomers of astaxanthin. According to Liu and Osawa [15], differences in the astaxanthin structure produce different biological effects. Astaxanthin has a significantly higher antioxidant capability compared to other carotenoids such as lutein, lycopene, zeaxanthin, canthaxanthin, α- carotene and b-carotene [16]. Depending on the source, astaxanthin can be found either as natural or synthetic. Natural astaxanthin can be obtained biosynthetically using yeast or microalgae, where conversely, synthetic astaxanthin can only be chemically produced [17]. Because of this, natural astaxanthin has been approved as a food additive in Europe, the United States and Japan. On the other hand, synthetic astaxanthin, due to the potential hazard of residual organic solvents used in its synthesis and the different biological activity of its isomers, is mainly used for aquaculture and animal nutrition [18]. Indeed, the use of synthetic astaxanthin is common practice for improving skin coloration in fish such as Atlantic salmon (*Salmo salar*) and *Pagrus* sp. [19], and it has also been shown to increase coloration in rainbow trout fillets [20]. Despite the wide use of artificial astaxanthin in fish nutrition, the production technology for natural astaxanthin has been improved, utilizing the necessary resources in a more sustainable way, allowing astaxanthin produced from algae (*Haematococcus pluvialis*) and yeast (*Phaffia rhodozyma*) to be more beneficial, in terms of utilization, for the cultivated fish. Previous studies used astaxanthin from algae *H. pluvialis* in red seabream nutrition up to ~70 mg kg^−1^ feed [21,22]. No previous studies found used yeast astaxanthin from *P. rhodozyma* in red seabream nutrition [19]. Furthermore and according to authors knowledge at the time, the comparison between natural astaxanthin sources in red seabream external coloration was never examined before. Hence, selecting the most promising source of astaxanthin and improving the supplementation level and feeding duration would benefit the overall red seabream production by reducing the production cost.

The aims of the present study were (i) to detect the astaxanthin source with the best efficacy in coloration for this species and (ii) to determine its optimal inclusion levels in red seabream feed based on the performance, skin coloration and physiological parameters of the fish. To this end, two experiments were conducted. In the first experimental trial, different sources of astaxanthin were tested with the same inclusion rate in the feed to identify the source with the best results in external coloration of the red seabream. Then, the second experimental trial was performed, where different levels of inclusion in the feeds of the best astaxanthin source, from the first trial, were tested. Hence, the best performing inclusion rate of astaxanthin source in relation to the feeding duration was identified, suggesting a best feeding practice (cost/effectiveness) in red seabream culture.

## 2. Materials and Methods

### 2.1. Experiment 1: Evaluating the Source of Astaxanthin

#### 2.1.1. Experimental Design

Healthy red seabream (*Pagrus major*) juveniles, produced under intensive cultivation conditions at the experimental facilities of the R&D department of Avramar Aquaculture S.A. (previously Nireus Aquaculture S.A., Koropi, Greece) were used. Until the start of the trial, fish were fed with a commercial red seabream diet with no astaxanthin supplementation. When fish average weight (±SD) reached 31.0 ± 2.0 g, nine 140 L cylinder–conical polyester tanks were prepared, and each tank accommodated 20 fish (180 fish in total). Tanks were in a closed water recirculation system with an average temperature of 18.2 ± 0.4 °C and dissolved oxygen > 6.3 mg L^−1^. The concentrations of NH_3_^−^, NO_2_^−^, and NO_3_^−^ were maintained at <0.05 mg L^−1^, <0.0005 mg L^−1^ and <0.05 mg L^−1^, respectively. The photoperiod cycle was set at 12L:12D under LED light with an intensity of 450 lux and temperature of 4000 K, which is a standard practice in Avramar’s commercial hatcheries.

Three experimental diets, supplemented with 100 mg kg^−1^ astaxanthin from three different sources, namely from *Haematococcus pluvialis*, *Phaffia rhodozyma*, and synthesized from chemicals (thereinafter named HM, PH and AS groups, respectively), were formulated (Table 1). This concentration was used since it is the maximum allowed level of inclusion in fish feeds according to Commission Implementing Regulation (EU) 2015/1415. The incorporation of the different astaxanthin sources in the experimental diets was achieved by reducing the wheat meal. Feeds were produced by a Clextral (Firminy, France) EV025 A107 extruder and a vacuum oil coater by Dinnissen (Sevenum, The Netherlands) Pegasus^®^ R&D Mixer—PG-10VC lab. Feeds were stored for 10 days in a dry room with a stable temperature of 21 ± 0.5 °C until their use (10 days). Fish were hand fed two times per day until satiation, for six days per week, for three months. Any uneaten feed was collected after each feeding and removed from the tanks in order to have a highly accurate calculation of FCR based on fish consumed feed only.

#### 2.1.2. Samplings

The experiment lasted three months, during which 3 samplings took place at 30, 60 and 90 days. Weight and standard length were determined at the beginning and at the end of the trial for all fish. Moreover, at the start of the trial, 20 additional individuals were anesthetized (200 ppm Ethylene glycol monophenyl ether, Merck 807291, Merck KGaA, Darmstadt, Germany) and sacrificed, and samples were taken to be used as a negative control (NC group, fish that never had astaxanthin before). Blood samples from the caudal vessel using heparinized syringes were collected at the beginning from all the fish in the NC group (day 0, *n* = 20 fish), and at the end of the trial (day 90) from 10 randomly selected fish per tank (*n* = 30 fish per treatment) after one day of fasting (24 h). Plasma, within 10 min after blood collection, was separated with centrifugation at 2500× *g* for 10 min and stored at −20 °C until further analysis. Similarly, skin samples and scales were collected at the beginning (NC group) and at the end of the trial from 5 randomly selected individuals per tank (*n* = 15 fish per treatment). For the determination of melanin content, skin samples were collected from the dorsal area of the fish and were stored in vials containing 95% ethanol [1], whereas scales for the quantification of the area covered with melanophores and erythrophores/xanthophores were collected and stored in saline solution. Colorimetric parameters of the skin were determined in all sampling points from all available fish using a portable colorimeter (Chroma meter Cr-400, Konica Minolta, Tokyo, Japan).

### 2.2. Experiment 2: Optimizing Astaxanthin Dose from Haematococcus pluvialis

#### 2.2.1. Experimental Design

New fish were provided from the same facility as Experiment 1. When fish reached a body weight ± SD of 32.0 ± 3.0 g, 280 fish were randomly distributed to 14 tanks (20 fish per tank) in the same closed recirculating system as Experiment 1 (four treatments with three replicate tanks and control with two replicate tanks). The average water temperature was 20.8 ± 1.3 °C, and dissolved oxygen was >6.2 mg L^−1^. The concentrations of NH_3_^−^, NO_2_^−^, and NO_3_^−^ were maintained at <0.05 mg L^−1^, <0.0005 mg L^−1^ and <0.05 mg L^−1^, respectively. Photoperiod cycle and light conditions follow the same protocol as Experiment 1.

The following five experimental diets were formulated: control (A0) with no astaxanthin addition and four diets with different concentrations of astaxanthin coming from *Haematococcus pluvialis* (20, 60, 80, and 100 mg kg^−1^) (Table 2). The same feeding protocol as Experiment 1 was followed.

#### 2.2.2. Samplings

The experiment lasted 60 days, during which three samplings took place at 0, 30 and 60 days. All fish were anesthetized as mentioned before, and the weight and standard length were measured to calculate growth factors. Fish color determination measurements took place on the 30th and 60th days of the trial using the same portable colorimeter as previously described. Scales and skin samples were also sampled accordingly for the chromatophores analysis. At the end of the trial, blood samples from 5 randomly selected fish per tank (*n* = 15 per treatment; *n* = 10 for the control group) were collected as previously mentioned (after one day of fasting). Plasma was separated within 10 min after blood collection with centrifugation at 2500× *g* for 10 min and stored at −20 °C until further analysis.

### 2.3. Growth Performance

Fish were left unfed for one day prior to samplings days, and all fish were weighed and leisured individually. Then, the following performance indices were calculated:

- Specific Growth Rate (SGR) % = {(LnMWfin − LnMWint)/days} × 100; MWfin: final mean weight, MWint: initial mean weight
- Total (cumulative) feed Conversion Rate (FCR) = feed fed kg/fish weight gain kg
- Condition factor (CF) = ((Fish body weight g/(Length cm^3^)) × 100
- Survival % = 100 − [(mortalities/initial stocking number) × 100]

### 2.4. Color Measurements

A portable colorimeter (Chroma meter Cr-400, Konica Minolta, Tokyo, Japan) was used to determine skin color. The color measurements were performed in two areas on fish skin, one at the dorsal area just below and at the start of the dorsal fin (D1) and one at the ventral area at the end of the pectoral fin (V1). The method used to measure color was based on the L*, a*, b* system proposed by the CIE (International Commission on Illumination). This system converts the perceived color spectrum information into the three parameters L*, which corresponds to lightness, a* for red and green, and b* for blue and yellow. After these measurements, the hue (H°ab) and the saturation (Chroma, C*ab) were determined using the following equations [23]:

For the Cab*:

C*ab = sqrt (a* 2 + b* 2)

The following cases are distinguished for the determination of factor H°ab:

- H°ab = tan^−1^ (b*/a*) for a* > 0 and b* ≥ 0
- H°ab = 180° + tan^−1^ (b*/a*) for a* < 0 and b* ≥ 0
- H°ab = 180° + tan^−1^ (b*/a*) for a* < 0 and b* < 0
- H°ab = 360° + tan^−1^ (b*/a*) for a*> 0 and b* < 0

### 2.5. Determination of the Melanin Content in Skin and Scales

The determination of the melanin content in the skin samples collected from the dorsal area of the fish during the initial and final sampling was based on the method developed previously [24,25].

The samples were first removed from the 95% ethanol in which they were kept until analysis and rinsed with deionized water to remove ethanol residues. They were then placed in 30 mL of 1% hydrochloric acid (HCl) in a 60 °C water bath for one hour. Hydrochloric acid led to decalcification of the samples. They were then repeatedly rinsed with deionized water and placed in a NaOH 0.2% solution at boiling temperature for one hour to dissolve the tissue and extract the melanin. Subsequently, the solution was filtered, and the absorbance of the sample was obtained using a spectrometer at 340 nm. The absorbance of each sample was converted to melanin concentration (expressed as μg mm^−2^) based on a standard curve of melanin prepared by dissolving pure melanin (from *Sepia officinalis*, Merck M-2649, Merck KGaA, Darmstadt, Germany) in a solution of 0.4 gr NaOH, 10 mL H_2_O, and 10 μL H_2_O_2_ 3% and the size (area) of the skin sample used.

### 2.6. Determination of the Area Covered from Melanophores and Count of Erythrophores/Xanthophores in Scales

The scales were observed with a stereoscope (Nikon SMZ645, Tokyo, Japan), and pictures were taken with a camera (Nikon Coolpix P6000, Tokyo, Japan) to detect the presence of melanophores, while erythrophores/xanthophores were observed using an optic microscope (Optika B500-TPH, Bergamo, Italy) with attached camera at 40× magnification. For the processing of the scales, the software Image J (ImageJ, 1.52, Bethesda, MD, USA) was used. The percentage % of the area covered from melanophores was determined at squares of specific size 150 × 150 pixels, randomly taken from the area of the scales that contained dermis. For the erythrophores/xanthophores, 4 random areas of the scale that contained dermis were photographed, and the total number of cells was counted.

### 2.7. Analytical Procedures

The levels of glucose, total cholesterol, triglycerides, and total proteins in the plasma were determined using commercially available enzymatic colorimetric assays (Biosis, Athens, Greece). The activity of the enzymes aspartate transaminase (AST) and alanine aminotransferase (ALT) were determined from the plasma with procedures of a commercial kit (Biosis, Athens, Greece). The concentration of cortisol in the plasma samples was determined by a commercial ELISA assay (Neogen, Lansing MI, USA), which was previously evaluated for use in the species [26]. Superoxide Dismutase (SOD) activity and Glutathione Peroxidase (GPx) were measured by a commercial kit (Cayman, Ann Arbor MI, USA).

### 2.8. Statistical Analysis

The statistical analysis of the results was performed with the software SPSS v.30 (Statistical Package for the Social Sciences, SPSS Inc., Chicago IL, USA). Normality (Shapiro–Wilk test) and homogeneity of variance were assessed, and data were log-transformed where necessary. Non-normally distributed data, even after transformation, were analyzed using the Kruskal–Wallis test. Results are presented as mean ± standard deviation (SD). Two-way Analysis of Variance (ANOVA) was used to test statistical differences between dietary groups Treatment and Sampling as factors for Experiment 1 and Treatment and Sampling as factors for Experiment 2. The statistical analysis for the Hue cyclical variable was performed with the Watson–Williams test for circular data using the Oriana software v4.02 (Kovach Computing Services, Anglesey, UK). For the physiological data, one-way nested ANOVA was performed to test statistical differences between different dietary groups. In all analyses, cases where statistically significant differences were observed were tested with Tukey’s test and compared for a significance level of 0.05.

## 3. Results

### 3.1. Experiment 1: Evaluating the Source of Astaxanthin

#### 3.1.1. Fish Growth Performance

Red seabream fed with HM astaxanthin had a significantly higher final weight compared to PH and was similar to AS (F(2, 6)= 5.481, *p* = 0.044). No other growth parameters showed any statistically significant differences among the experimental groups (Table 3). The mortalities registered during the trial period were attributed as random without an evident cause.

#### 3.1.2. Skin Coloration

**Figure 1:**
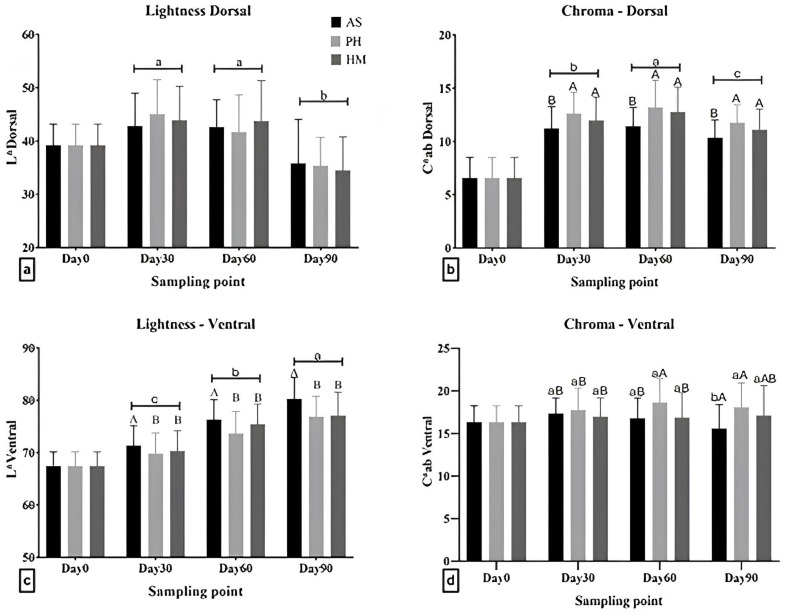
Lightness (L*) and chroma (C*ab) in the dorsal (**a**,**b**) and ventral (**c**,**d**) skin area of red seabream fed with feed containing astaxanthin from different sources. Values are reported as mean ± SD. Samplings were performed on day 30, 60 and 90. Different lowercase letters indicate statistical differences between sampling points within each group; different uppercase letters indicate differences between groups within each sampling point (*p* < 0.05). Data from Day 0 were not used in the statistical analysis due to the experimental design and were presented only for qualitative comparisons. AS: synthetic astaxanthin; PH: astaxanthin from *Phaffia rhodozyma*; HM: astaxanthin from *Haematococcus pluvialis*.

The source of astaxanthin contained in the experimental diets affected the external coloration, with significant differences observed between the different treatments for the color parameters (L*, C*ab, H°ab) (Figure 1 and Table 4).

Skin brightness in the dorsal area of the fish was not affected by the source of astaxanthin (F(2, 510) = 0.098; *p* = 0.907) but was affected by the sampling period (F(2, 510) = 87.93; *p* < 0.001) (Figure 1a). Specifically, on day 90, the lightness of the dorsal area was lower than day 30 and 60 in all groups (*p* < 0.05). In the ventral area, significant differences between groups receiving different sources of astaxanthin were observed (F(2, 510) = 17.75; *p* < 0.001); in particular, they are higher in fish fed with synthetic astaxanthin than the other two groups. The factor sampling also had a significant effect (F(2, 510) = 157.4; *p* < 0.001), increasing at each sampling (Figure 1c).

Regarding the chroma (C*ab) in the dorsal area, it was shown that both the treatment (F(2, 510) = 25.17; *p* < 0.001) and the sampling (F(2, 510) = 20.60; *p* < 0.001) affected the results. Specifically, fish that were fed with feed containing synthetic astaxanthin had lower chroma in the dorsal area than fish fed with both types of natural source (Figure 1b). In terms of sampling, chroma reached maximum levels on day 60, being lower on day 30 and again lower on day 90. Still, all samplings had approximately 1.6 to 2.0 higher chroma than day 0. Finally, regarding chroma at the ventral area, a significant interaction was observed between treatment and sampling points (F(4, 510) = 3.036; *p* = 0.017) (Figure 1d). Significant differences were observed between fish fed with PH compared to the other two feed types at day 60 and between AS and PH at day 90 (Figure 1d). Differences between samplings were only observed in the group fed with AS between day 30 and day 90 (Figure 1d).

**Figure 2:**
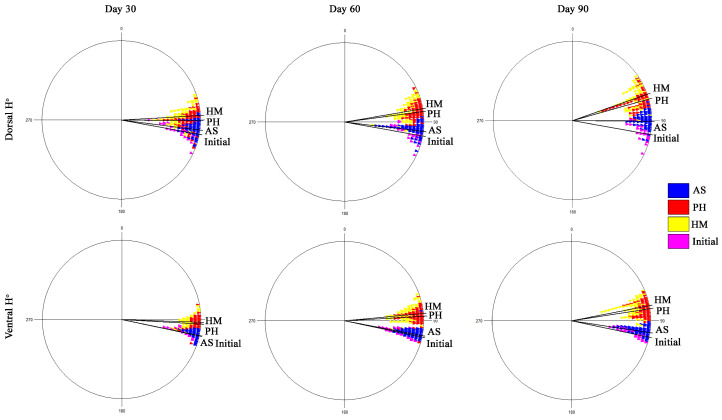
Hunter diagram illustrating hue mean values of ventral and dorsal skin areas for all treatments at 30, 60 and 90 days. AS: synthetic astaxanthin, PH: *Phaffia rhodozyma*, HM: *Haematococcus pluvialis* and the values from the initial sampling day.

The results obtained from the determination of the color parameter hue (H°ab) of the fish’s skin showed that a change in the shade towards the desired pinkish coloration was observed in all three experimental groups in both dorsal (F(8, 18) = 74.124; *p* < 0.001) and ventral (F(8, 18) = 167.16; *p* < 0.001) areas (Table 4). However, the experimental groups that were fed with diets containing natural astaxanthin (PH and HM groups) showed a more significant and faster improvement of coloration than the synthetic astaxanthin group (Table 4). According to the circular color model used, purple–red shades are found at 0° while yellow, green, and blue shades are, respectively, found at 90°, 180° and 270°. The hue of the fish of the experimental group for which the microalgae *Haematococcus pluvialis* was used as a source of astaxanthin approached, to a greater extent, the pink hue compared to the other experimental groups, with an average value of the hue parameter being 70.5° and 78.9° for the dorsal and ventral regions, respectively, 90 days from the start of the experiment (Figure 2).

#### 3.1.3. Pigment Cells

The results from the analysis of images obtained from a stereoscope to assess the area covered by melanophores showed no statistically significant differences between the experimental groups. The coverage percentages (mean ± SD, *n* = 3 per tank; *n* = 3 tanks per treatment) of melanophores in the dorsal region were 9.2 ± 4.5%, 9.2 ± 3.4%, and 9.4 ± 4.0% for the fish fed with the diets AS, PH and HM, respectively, while for the ventral region, they were 3.7 ± 1.9%, 3.6 ± 2.1%, and 4.1 ± 1.2%, respectively.

The presence of erythrophores and xanthophores on the scales was also observed; however, it was not possible to accurately distinguish them and determine the coverage area. For this reason, only the total number of erythrophores and xanthophores was determined, which did not differ statistically significantly between groups. In the dorsal region, the total number (mean ± SD, *n* = 3 per tank; *n* = 3 tanks per treatment) of erythrophores/xanthophores per scale was 32.2 ± 6.0, 34.3 ± 8.5 and 33.8 ± 6.9 for the fish fed with the diets AS, PH and HM, respectively, while, for the ventral region, it was 25.2 ± 7.6, 23.3 ± 6.0 and 24.8 ± 6.2, respectively.

#### 3.1.4. Stress Indicators, Biochemical Parameters, and Antioxidant Capacity

No statistically significant differences were observed in the mean concentrations of glucose and total cholesterol in the plasma of fish between the different experimental groups at day 90 (Table 5). In all groups, however, a statistically significant (*p* < 0.05) decrease in mean glucose and total cholesterol concentrations was observed compared to the initial concentrations at Day 0.

Total proteins, triglycerides, and liver enzymes AST, ALT did not show statistically significant differences between groups and initial and final sampling (Table 5). Plasma cortisol, an indicator of fish stress, and the antioxidant enzyme SOD measured in plasma did not show statistically significant differences between groups and between initial and final sampling.

### 3.2. Experiment 2: Optimizing Astaxanthin Dose from Haematococcus pluvialis

#### 3.2.1. Fish Growth Performance

Red seabream equally accepted the inclusion of astaxanthin in feed at different concentrations, showing no significant (*p* > 0.05) impact on growth parameters and feed intake (Table 6). The mortalities registered during the trial period were attributed as random without an evident cause.

#### 3.2.2. Skin Coloration

**Figure 3:**
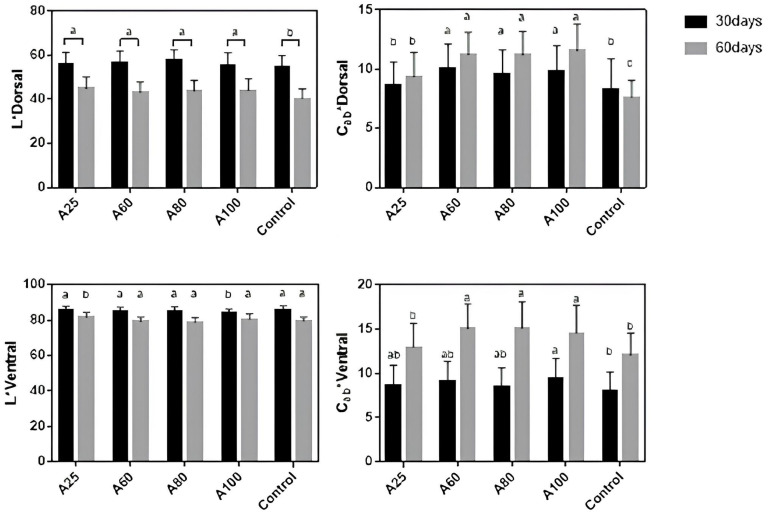
Values of lightness (L*) and chroma (C*ab) in the dorsal and ventral skin areas of red seabream fed at different natural astaxanthin concentrations. Data are reported as mean ± SD (*n* = 20; 60 fish per treatment). Samplings were performed on day 30 and day 60. All means between groups differed significantly from each sampling (*p* < 0.05). Letters only compare values between the groups within each sampling; means with common letters denote no significant differences (*p* < 0.05).

Significant differences between the color of the dorsal and ventral areas of the fish were observed across all experimental diets, with the dorsal section being notably more pigmented. A marked dorso–ventral gradient of color attributes (L*, C*ab, and H°ab) related to astaxanthin concentration was observed, with lighter ventral skin than the dorsal area. Two-way ANOVA indicated a significant difference between sampling days and treatments for all parameters (*p* < 0.05). Values for lightness in the dorsal area did not present a statistically significant interaction among sampling and treatments (Figure 3). Lightness values for astaxanthin diets differed significantly from the control during the trial on the dorsal area. Samples at day 60 showed a decrease of approximately 30% in lightness compared to day 30, ranging from 52.8–56.2 to 37.5–42.0, with A25 having the highest value among astaxanthin diets. In contrast to lightness, the highest concentration of astaxanthin (A60, A80, A100) affected the chroma values significantly in the dorsal and ventral areas, with no difference among them (*p* > 0.05). A sharp rise in the chroma of the ventral area was registered from 30 to 60 days for all diets, increasing from 8.9–9.4 to 14.6–15.1 in A60, A80, and A100 concentrations (indicative photos of fish S1_A0, S2_A25, S3_A60, S4_A80, S5_A100 can be found in the Supplementary Materials).

The colorimetric analysis showed the effect of natural astaxanthin on the reddish color pigmentation of the red seabream. More specifically, hue values measured in this trial reflect a positive impact on reddish coloration from A60 up to A100 in the dorsal area, with no significant variance between them (Table 7). According to the CIE concept, a* represents the redness, and b* is the yellowish in the skin. A significative increment in both areas and sampling was obtained for a* and b*. The concentrations of A60, A80, and A100 expressed similarly high values for redness and yellowish in comparison to ventral greenish color (-a*).

**Figure 4:**
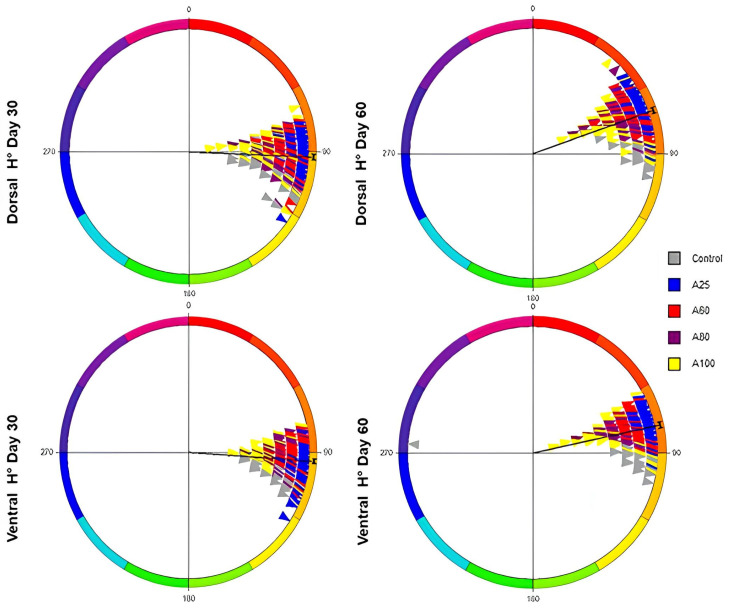
Hunter diagram illustrating the mean values of hue of ventral and dorsal skin areas for all treatments at 30 and 60 days. A control feed (A0) with no astaxanthin addition and four feeds with different concentrations of astaxanthin, coming from *H. pluvialis* 20, 60, 80, and 100 mg kg^−1^ as A20, A60, A80, and A100, respectively, were used.

Hue results are displayed in the hunter diagram [27], representing the a*-b* color space (0–360°). Hue values near to 0° are close to red color, while 90° means yellow color (Figure 4). Values for all diets and sampling days were predominantly ranged in the red/yellow color spectrum, except the ventral area at day 60, which exhibited a green hue. An apparent shift from yellow to reddish skin color in astaxanthin diets was observed, in line with the increment of erythrophores and hue values at the end of the trial.

#### 3.2.3. Pigment Cells

The melanin skin deposition in the dorsal area at the end of the trial did not differ between the astaxanthin diets and control (Table 8). The analysis of ventral melanin registered values below the detection limit. Additionally, two types of pigment cells were measured in the fish. Melanophores, identified as the more prominent and darker cell pigment, ranged from fully aggregated to dispersed as branches and erythrophores/xanthophores and were recognized and counted as the small and concentrated red/yellow granules.

A statistically significant difference in sampled melanophores areas (*p* < 0.05) was observed (Table 8). The dorsal skin section displayed a proportion of melanophores covering 1.2–6.1% over the 60 days, while the ventral area showed a coverage of 0.00–0.03%. The percentage of melanophores in the dorsal area did not present a significant variance among the experimental diets and control except for A25 values, which showed the lowest coverage at Day 60 (*p* < 0.05). An increase in darkening of the dorsal fish skin was observed from day 30 to day 60, doubling their coverage area in almost all diets.

#### 3.2.4. Stress Indicators, Biochemical Parameters, and Antioxidant Capacity

The biochemical parameters of total cholesterol and glucose did not present a significant difference between the treatments (Table 9). However, triglyceride concentrations in fish plasma fed with astaxanthin were three times higher than the control values. Total protein concentrations also differed between the experimental diets, with higher values for fish supplied with A100 compared to A60, A80, and the control. Hormonal and antioxidant parameters, measured as cortisol and SOD, GPx, respectively, were not altered by the astaxanthin concentration present in the feed.

## 4. Discussion

This study demonstrates that the source of astaxanthin significantly impacts the growth performance and pigmentation of cultivated fish. Specifically, in Experiment 1, fish fed feed containing astaxanthin from the algae *Haematococcus pluvialis* exhibited greater final weights than those receiving astaxanthin from the yeast *Phaffia rhodozyma*. However, no significant weight differences were observed between fish fed synthetic astaxanthin and the other groups. Experiment 2 revealed that the different inclusion levels of *H. pluvialis* astaxanthin in the experimental feeds produced no significant effects on growth performance, suggesting qualitative differences among astaxanthin sources rather than a dose-dependent effect. These findings align with previous studies in *Pagrus* sp. [2,5,11,21,22] and other fish, such as Atlantic salmon (*Salmo salar*) [28,29,30] and rainbow trout (*Oncorhynchus mykiss*) [31]. Thus, the present study concludes that, even at the higher level allowed by EU regulation, which is an inclusion level of 100 mg kg^−1^ astaxanthin in the fish feed, growth promotion cannot be achieved. On the other hand, there are studies that reveal a positive correlation between the astaxanthin supplementation in the feeds and either growth or survival or both in fish, such as red bream (*Pagrus pagrus*) [10,32], Atlantic cod (*Gadus morhua*) [33] and Nile tilapia (*Oreochromis niloticus*) [34]. The above-mentioned studies verify the importance of astaxanthin supplementation in the feeds for aquatic animal physiology. Depending on the species, the feeding duration (up to 120 days) and the level of astaxanthin supplementation (up to 1 g kg^−1^) can enhance physiological functions and subsequently enhance the nutrient utilization [31,32,33,35,36].

A key challenge in the production of red seabream is achieving optimal coloration. This problem has the following two components: first the hypermelanosis, i.e., the dark color of the skin, and second, the dyspigmentation, which refers to the loss of the red/pink natural color of the fish. Fish skin coloration is regulated by a complex interplay of intrinsic factors, including but not limited to neuroendocrine and cellular responses, stress physiology, and genetic background, as well as extrinsic influences such as feed composition and rearing conditions [12]. Besides the fact that skin brightness is related to physiological and rearing conditions, it appears that choosing a lighter background in the rearing tanks/cages, rearing temperature close to 19 °C, and keeping a low intensity of blue light can increase the skin lightness [1,11,37]. Additionally, unfavorable rearing conditions can reduce the skin brightness as a protective mechanism and compensate for the stress [1,24,37]. Based on the findings of Experiment 1, skin brightness (measured as L*) was not affected by the three different sources of astaxanthin. This is in agreement with previous studies on *Pagrus pagrus* [2,24,35], which suggested that the astaxanthin source cannot affect skin lightness. In agreement, Experiment 2 concluded that there was no dose dependency between astaxanthin and skin brightness, especially in the dorsal region.

With regards to dyspigmentation, Experiment 1 revealed that the coloration of red seabream was enhanced with the feed containing astaxanthin from the *H. pluvialis*, followed by the *P. rhodozyma* and synthetic astaxanthin. Consequently, Experiment 2 exclusively used *H. pluvialis* as the astaxanthin source. The algae *H. pluvialis* astaxanthin consists of 3S,3′S isomer containing 70% mono-ester form, 25% di-ester form and 5% of free form (not esterified) [38]. The yeast *P. rhodozyma* astaxanthin consists of 3R,3′R isomer in 97% free (non-esterified) form, with the remaining 3% in other forms [39]. The artificially synthesized astaxanthin consists of 3S,3′S, 3R,3′S and 3R,3′R isomers in a proportion ratio of 1:2:1, respectively, containing only the free form [40]. According to Doolan et al. [5] and Chatzifotis et al. [22], the esterified and specifically the di-ester form of astaxanthin can be found in the skin of red seabream, which support the present findings. Furthermore, the 3S,3′S isomer of astaxanthin appears to have increased beneficial biological effects not only in fish but in humas as well, compared to the remaining isomer forms [39]. Despite no beneficial effects of the remaining astaxanthin isomer forms, no harmful incidents have been reported. Additionally, the present findings agree with an earlier study using the same species, where natural sources of astaxanthin (*P. rhodozyma* and *Paracoccus* sp.) increased red coloration compared to the artificially synthesized astaxanthin [41]. In detail, the best results came from the *Paracoccus* sp., followed by the *P. rhodozyma* and then the artificially synthesized astaxanthin, and they were related to the content of the 3S,3′S astaxanthin isomer, which was present in the *Paracoccus* sp., similarly to the *H. pluvialis*.

It has been previously reported that improved redness coloration in red seabreams was achieved using astaxanthin inclusion rates between 36 and 72 mg kg^−1^ of feed [4,21,42]. Experiment 2, in which different levels of astaxanthin were tested, confirmed that skin redness measured as a* was affected even with the lower level of astaxanthin supplementation in the feed. Similarly, b* values were also increased, indicating that the yellowish color of the red seabream skin can also be affected by the inclusion of 60 mg kg^−1^ and above of astaxanthin in the feed. This agrees with previous studies that have found that astaxanthin can synthesize tunaxanthin, a yellow carotenoid which can be present in the red seabream skin, promoting the occurrence of xanthophores [21,43]. Under intensive farming conditions, the optimal level of astaxanthin supplementation together with the appropriate time of administration will not only ensure the desirable pigmentation of the red seabream skin but will also enhance the profitability of the production. Based on the findings of the present study, astaxanthin supplementation of 60 mg kg^−1^ feed from *H. pluvialis* for 60 days can attain the desirable color of red seabream. Among the tested sources of astaxanthin, *H. pluvialis* had the highest level of esterified astaxanthin concentration, which produced the desirable effect with lower inclusion rates compared to the other sources. Taking into consideration the public awareness for using natural sources to achieve the desirable color of the farmed red seabream [38], *H. pluvialis* turns out to be the most sustainable and profitable natural source of astaxanthin for the aquaculture industry [44].

The inclusion of astaxanthin in the feed seemed to affect some biochemical parameters but had little effect on the hormonal and enzymatic profile of the fish in both experiments. The impact on biochemical parameters varied between the two trials. Specifically, the inclusion of astaxanthin—regardless of its source—led to lower levels of glucose and cholesterol in Experiment 1 and to higher levels of triglycerides in Experiment 2. In the latter experiment, increased levels of total proteins were also observed in fish fed with the highest and lowest levels of astaxanthin compared to the rest of the groups. In all cases, data were similar to previously published data for this species [3,45,46,47,48,49,50]. In general, dietary astaxanthin has been shown to alter lipid metabolism and glucose regulation in mammals [50] and also in fish [51]. While no studies have specifically examined the biochemical effects of astaxanthin in red seabream, research on other fish species has reported reductions in glucose, cholesterol, and triglycerides, such as in Asian seabass (*Lates calcarifer*) [52] and rainbow trout (*Oncorhynchus mykiss*) [53], while others found no effects, such as in pufferfish (*Takifugu obscurus*) [54] and yellow catfish (*Pelteobagrus fulvidraco*) [55]. The observed reduction of glucose and cholesterol levels in Experiment 1 is consistent with other previous studies and may have been a result of the inclusion of astaxanthin. On the other hand, to the best of our knowledge, no studies have reported an increase in the levels of triglycerides due to the consumption of astaxanthin, as was observed in Experiment 2. Based on this, we cannot conclude the factor causing the elevation of triglycerides compared to control in this experiment, and further research is needed.

Astaxanthin is known to have antioxidant properties [16]; it was hypothesized that different sources and inclusion levels of astaxanthin might modulate stress responsiveness in red sea bream. However, no differences in cortisol were observed between astaxanthin-fed and control fish in both experiments, indicating that the inclusion of astaxanthin has no effect on the regulation of this hormone. In both experiments, the levels of cortisol were comparable to previously published data for this species [45,56,57,58,59] but higher than other studies [3,46,47,49,50,60,61,62]. In Experiment 1, cortisol levels did not differ between groups fed astaxanthin from different sources, indicating that all forms were well tolerated in terms of stress response. In Experiment 2, the inclusion of astaxanthin in high concentrations (A80, A100) resulted in significantly higher levels of cortisol compared to A60, with the A25 and the control fish not differing from the other groups. Until now, there has been little information about the effect of astaxanthin on circulating cortisol levels in fish. The few published studies show that these effects are species-specific ranging from no effect in yellow catfish [55], to reducing cortisol in high astaxanthin doses in rainbow trout [53], and reducing cortisol in all examined doses in Asian seabass (*Lates calcarifer*) [52].

The strong antioxidant properties of astaxanthin have led to its various applications in the food industry [63,64]. Due to its chemical structure, it has the ability to non-enzymatically scavenge Reactive Oxygen Species (ROS) produced by chemical reactions taking place in the cells. According to theory, the reduction of ROS by astaxanthin should reduce the presence of antioxidant enzymes in the animals, such as, for example, superoxide dismutase, glutathione peroxidase and catalase [64]. In fish, this has been observed in one study in characin (*Hyphessobrycon eques*) [65]. However, in practice, this has not been clearly proven in other studies in fish. Similar to the results presented here, studies on rainbow trout showed that, although fish fed astaxanthin indeed had reduced levels of ROS, the levels of SOD and GPx were not affected [20,66,67]. Similar results were found in yellow catfish [55]. On the contrary, other studies have reported increased SOD and GPx activity in rainbow trout [53] and coral trout (*Plectropomus leopardus*) [68] following astaxanthin supplementation.

Apart from the antioxidant enzymes, astaxanthin has been reported to positively influence the activity of the hepatic enzymes alanine (ALT) and aspartate (AST) aminotransferases. Specifically, studies on fish species have shown that both enzymes show lower activity in fish fed astaxanthin [38,53,54,65,69,70]. In the present study, no effects of dietary astaxanthin were observed. The levels of both ALT and AST were similar to most previously published studies for the species [46,57,59], with the exception of AST being higher than the studies of Hwang et al. [47] and Jeong et al. [48]. Since these hepatic enzymes are indicators of liver function, the fact that there were no effects of dietary astaxanthin and that the data of fish fed astaxanthin were similar to previously published studies indicates that it is safe for fish health and function to include astaxanthin of all sources examined.

## 5. Conclusions

The present study provides a comprehensive evaluation of the effects of dietary astaxanthin in red seabream by integrating physiological, biochemical, stress-related, and pigmentation responses across different sources, inclusion levels, and feeding durations. Across all treatments, astaxanthin supplementation posed no detectable risk to fish health, as evidenced by the absence of adverse effects on biochemical, enzymatic, and stress indicators, supporting its safe use in red seabream diets at inclusion levels up to 100 mg kg^−1^. On the other hand, although dietary astaxanthin is known to act as a non-enzymatic antioxidant, no differences in the activities of the antioxidant enzymes SOD and GPx as well as the hepatic enzymes ALT and AST were observed. However, since the total amount of ROS or the total antioxidant capacity were not measured in this study, it is not possible to fully evaluate the function of astaxanthin as an antioxidant in this species.

With respect to product quality, this study clearly demonstrates that skin coloration is influenced by astaxanthin source, inclusion level, and feeding duration. Astaxanthin derived from *Haematococcus pluvialis* consistently resulted in superior pigmentation compared to synthetic and yeast-derived sources, while an inclusion level of 60 mg kg^−1^ was sufficient to achieve coloration comparable to higher dosages.

Overall, the findings indicate that algal-derived astaxanthin at 60 mg kg^−1^ represents an effective and efficient strategy to optimize skin coloration in red seabream while maintaining physiological homeostasis. To strengthen these conclusions, future research should evaluate antioxidant capacity using integrative oxidative stress markers and extend feeding trials to market-size fish (600–800 g) to assess the persistence and commercial relevance of pigmentation responses, including effects on skin lightness and consumer-relevant quality traits.

## Figures and Tables

**Table 1:** Formulation and proximate composition of experimental diets used in Experiment 1.

Ingredients (% DM)	AS	PH	HM
Fish meal 67% ^1^	28.10	28.10	28.10
Fish oil ^2^	8.00	8.00	8.00
Soya Protein Concentrate	15.30	15.30	15.30
Corn gluten meal	20.10	20.10	20.10
Wheat gluten meal	6.00	6.00	6.00
Wheat meal	11.70	11.50	11.50
Plant premix ^3^	1.00	1.00	1.00
Vitamin & mineral premix ^4^	1.80	1.80	1.80
Monocalcium phosphate	7.00	7.00	7.00
Soya lecithin	0.20	0.20	0.20
Taurine	0.70	0.70	0.70
Synthetic astaxanthin 10% ^5^	0.10		
*Phaffia rhodozyma* 3.2%AST ^6^		0.30	
*Haematococcus pluvialis* 3.2%AST ^7^			0.30
Sum	100.00	100.00	100.00
**Proximate composition %**	-	-	-
Dry matter	96.0	95.4	94.8
Moisture	4.0	4.6	5.2
Crude proteins	49.6	49.8	49.1
Crude fats	13.2	14.1	13.2
Crude fibers	0.87	0.98	0.97
Total ash	16.1	16.7	16.0

HM, PH and AS are experimental diets with the astaxanthin inclusion from *Haematococcus pluvialis*, *Phaffia rhodozyma* and chemically synthesized, respectively. ^1^ FF Skagen, Skagen, Danish. ^2^ ΝORSILDMEL, Bergen, Norway. ^3^ FF2.0^®^, Greenfeedcycles, San Lazzaro di Savena, Italy. ^4^ Choline: 90.000 mg/kg, inositol: 15.700 mg/kg, VIT C: 20.300 mg/kg, VIT A: 300.000 IU/kg, VIT D3: 100.000 IU/kg, VIT E: 20.000 IU/kg, VIT K: 820 mg/kg, VIT B1: 300 mg/kg, VIT B2: 550 mg/kg, VIT B3: 1.800 mg/kg, VIT B5: 3.300 mg/kg, VIT B6: 900 mg/kg, VIT B7: 12 mg/kg, VIT B9: 300 mg/kg, VIT B12: 15 mg/kg, Cu: 450 mg/kg, Zn: 6.750 mg/kg, Mn: 1.200 mg/kg, Fe: 765 mg/kg, Se:14 mg/kg, I: 100 mg/kg. ^5^ Lucantin^®^ Red 10% astaxanthin concentration, BASF, Ludwigshafen, Germany. ^6^ BPAX-AL, 3.2% astaxanthin concentration (AST), Bioproton Pty Ltd., Acacia Ridge, Australia. ^7^ AstaPure^®^, 3.2% astaxanthin concentration (AST), Algatech, Ketura, Israel.

**Table 2:** Formulation and proximate composition of experimental diets used in Experiment 2.

Ingredients %	A0	A25	A60	A80	A100
Fish meal 67% ^1^	32.40	32.40	32.40	32.40	32.40
Fish oil ^2^	8.30	8.30	8.30	8.30	8.30
Soya bean meal	15.00	15.00	15.00	15.00	15.00
Soya Protein Concentrate	6.90	6.90	6.90	6.90	6.90
Corn gluten meal	5.00	5.00	5.00	5.00	5.00
Sunflower meal	6.70	6.70	6.70	6.70	6.70
Rapeseed meal	6.70	6.70	6.70	6.70	6.70
Wheat meal	14.70	14.63	14.50	14.45	14.40
Plant premix ^3^	1.00	1.00	1.00	1.00	1.00
Vitamin and mineral premix ^4^	1.80	1.80	1.80	1.80	1.80
Monocalcium phosphate	1.00	1.00	1.00	1.00	1.00
Taurine	0.50	0.50	0.50	0.50	0.50
*Haematococcus pluvialis* 3.2%AST ^5^	0.00	0.07	0.20	0.25	0.30
Sum	100.00	100.00	100.00	100.00	100.00
**Proximate composition %**					
Dry matter	94.2	94.3	94.1	94.0	93.8
Moisture	5.8	5.7	5.9	6.0	6.2
Crude proteins	46.5	46.4	46.7	46.4	46.3
Crude fats	14.0	13.6	13.8	14.4	14.4
Crude fibers	2.7	2.9	2.6	2.7	2.7
Total ash	9.5	9.5	9.5	9.5	9.4

A0, A25, A60, A80 and A100 indicate the experimental feeds and the level of astaxanthin supplementation 0, 25, 60, 80 and 100 mg kg^−1^ of feed, respectively. Raw material levels are given in g per 100 g of total raw material mix (dry matter). The incorporation of the different *Haematococcus pluvialis* levels was conducted in the reduction of wheat meal. ^1^ FF Skagen, Skagen, Danish. ^2^ ΝORSILDMEL, Bergen, Norway. ^3^ FF2.0^®^, Greenfeedcycles, San Lazzaro di Savena, Italy. ^4^ Choline: 90.000 mg/kg, inositol: 15.700 mg/kg, VIT C: 20.300 mg/kg, VIT A: 300.000 IU/kg, VIT D3: 100.000 IU/kg, VIT E: 20.000 IU/kg, VIT K: 820 mg/kg, VIT B1: 300 mg/kg, VIT B2: 550 mg/kg, VIT B3: 1.800 mg/kg, VIT B5: 3.300 mg/kg, VIT B6: 900 mg/kg, VIT B7: 12 mg/kg, VIT B9: 300 mg/kg, VIT B12: 15 mg/kg, Cu: 450 mg/kg, Zn: 6.750 mg/kg, Mn: 1.200 mg/kg, Fe: 765 mg/kg, Se:14 mg/kg, I: 100 mg/kg. ^5^ AstaPure^®^, 3.2% astaxanthin concentration (AST), Algatech, Ketura, Israel.

**Table 3:** Fish growth performance parameters for Experiment 1 with statistical differences located only for final weight.

	AS	PH	HM	*p*-Value
Initial weight (g)	31.5 ± 1.9	31.6 ± 1.9	31.7 ± 1.9	0.870
Final weight (g)	70.6 ± 12.1 ^ab^	67.1 ± 12.8 ^b^	72.7 ± 12.7 ^a^	0.044
Total Biomass (g)	1341.8 ± 47.6	1319.4 ± 74.3	1284.8 ± 111.7	0.707
SGR	0.87 ± 0.03	0.82 ± 0.04	0.91 ± 0.03	0.061
FCR	1.22 ± 0.05	1.31 ± 0.06	1.25 ± 0.13	0.469
CF	3.49 ± 0.45	3.47 ± 0.49	3.41 ± 0.44	0.105
Survival%	95.0 ± 5.0	98.3 ± 2.9	88.3 ± 7.6	0.158

HM, PH and AS are experimental diets with the astaxanthin inclusion from *Haematococcus pluvialis*, *Phaffia rhodozyma* and chemically synthesized, respectively. Growth performance parameters i.e., SGR: Specific Growth Rate, FCR: Feed conversion Index, and CF: Condition Factor. Values are presented as mean ± SD. Different letters signify statistically significant differences.

**Table 4:** Values of hue (H°ab) in the dorsal and ventral skin area of red seabream fed with feed containing astaxanthin from different sources.

	**Dorsal**			**Ventral**		
Day 0	100.3 ± 6.0			102.0 ± 2.9		
**Treatment**	**AS**	**PH**	**HM**	**AS**	**PH**	**HM**
Day 30	97.2 ± 1.9 ^aA^	90.5 ± 1.3 ^aB^	85.5 ± 1.2 ^aC^	101.7 ± 0.8 ^aA^	93.3 ± 1.0 ^aB^	92.0 ± 0.6 ^aB^
Day 60	96.8 ± 0.2 ^aA^	82.1 ± 0.8 ^bB^	80.3 ± 2.3 ^bB^	100.8 ± 0.2 ^aA^	86.8 ± 1.2 ^bB^	84.8 ± 1.6 ^bB^
Day 90	90.3 ± 2.6 ^bA^	74.1 ± 1.2 ^cB^	70.5 ± 0.9 ^cC^	98.6 ± 1.1 ^bA^	81.3 ± 0.3 ^cB^	78.9 ± 0.6 ^cC^

Data are reported as mean ± SD (*n* = 20 per tank; *n* = 3 tanks per treatment). Samplings were performed on days 30, 60 and 90. Different lowercase letters indicate statistical differences between sampling points within each group (vertically); different uppercase letters indicate differences between groups within each sampling point (horizontally) (*p* < 0.05). Data from Day 0 were not used in the statistical analysis due to the experimental design and are presented only for qualitative comparisons. AS: synthetic astaxanthin; PH: astaxanthin from *Phaffia rhodozyma*; HM: astaxanthin from *Haematococcus pluvialis*.

**Table 5:** The effects of astaxanthin source on biochemical (glucose, cholesterol, triglycerides, total proteins), enzymatic (ALT, AST, SOD) and hormonal (cortisol) indicators.

	Day 0	Day 90		
Astaxanthin Source	Control	AS	PH	HM
Glucose (mmol L^−1^)	5.3 ± 1.0 ^a^	3.1 ± 0.8 ^b^	3.1 ± 0.9 ^b^	3.3 ± 1.0 ^b^
Cholesterol (mmol L^−1^)	2.8 ± 0.6 ^a^	1.3 ± 0.3 ^b^	1.3 ± 0.4 ^b^	1.4 ± 0.7 ^b^
Triglycerides (mmol L^−1^)	1.2 ± 0.9	1.3 ± 0.5	1.4 ± 0.6	1.6 ± 0.7
Total Proteins (g dL^−1^)	3.1 ± 0.4	3.1 ± 0.6	3.2 ± 0.4	3.0 ± 0.6
ALT (U L^−1^)	107 ± 124	220 ± 117	168 ± 140	151 ± 127
AST(U L^−1^)	264 ± 121	182 ± 135	250 ± 104	194 ± 140
SOD (U mL^−1^)	1.9 ± 1.1	2.1 ± 1.4	1.8 ± 1.4	2.3 ± 1.8
Cortisol (ng mL^−1^)	61.6 ± 42.0	64.9 ± 40.4	65.2 ± 44.1	60.1 ± 40.1

Different letters indicate statistically significant differences (*p* < 0.05) between the treatments within each indicator. AS: synthetic astaxanthin; PH: astaxanthin from *Phaffia rhodozyma*; HM: astaxanthin from *Haematococcus pluvialis*. Values are presented as mean ± SD.

**Table 6:** Growth performance and feed utilization of red seabream after being fed with the astaxanthin diets for 60 days.

	A25	A60	A80	A100	A0	*p*-Value
Initial weight (g)	32.0 ± 0.04	32.1 ± 0.07	32.0 ± 0.04	32.0 ± 0.03	32.0 ± 0.03	1.000
Final weight (g)	68.9 ± 3.9	69.9 ± 1.6	65.7 ± 0.9	69.8 ± 3.6	69.1 ± 0.8	0.097
Total Biomass (g)	1353.2 ± 34.2	1326.7 ± 39.1	1248.2 ± 83.3	1326.3 ± 56.5	1347.6 ± 22.9	0.386
SGR	1.28 ± 0.3	1.30 ± 0.1	1.20 ± 0.1	1.30 ± 0.3	1.28 ± 0.1	0.337
FCR	1.16 ± 0.07	1.10 ± 0.06	1.21 ± 0.1	1.17 ± 0.09	1.09 ± 0.04	0.389
CF	3.17 ± 0.03	3.18 ± 0.02	3.17 ± 0.10	3.18 ± 0.05	3.11 ± 0.10	0.216
Survival%	98.3 ± 2.9	95.0 ± 5.0	93.3 ± 5.8	93.3 ± 2.9	95.0 ± 0.0	0.596

A0, A25, A60, A80 and A100 indicate the experimental feeds and the level of astaxanthin supplementation 0, 25, 60, 80 and 100 mg kg^−1^ of feed, respectively. Values are presented as mean ± SD.

**Table 7:** Hue values of the dorsal and ventral skin area of red seabream fed with different astaxanthin concentrations.

	Dorsal		Ventral	
Treatment	Day 30	Day 60	Day 30	Day 60
A25	89.7 ± 9.4 ^b^	67.9 ± 8.7 ^b^	92.9 ± 27.6 ^bcd^	74.7 ± 6.4 ^bc^
A60	90.6 ± 9.8 ^b^	68.6 ± 8.4 ^b^	94.5 ± 35.2 ^bcd^	75.9 ± 7.2 ^d^
A80	91.9 ± 9.5 ^b^	67.7 ± 8.2 ^b^	93.2 ± 6.7 ^c^	76.1 ± 5.3 ^c^
A100	89.1 ± 10.2 ^b^	68.1 ± 10.7 ^b^	91.2 ± 16.7 ^d^	76.0 ± 9.0 ^bd^
A0	104.3 ± 8.6 ^a^	89.9 ± 6.3 ^a^	104.5 ± 4.4 ^a^	94.6 ± 21.6 ^a^

A0, A25, A60, A80 and A100 indicate the experimental feeds and the level of astaxanthin supplementation 0, 25, 60, 80 and 100 mg kg^−1^ of feed, respectively. Values are presented as mean ± SD (*n* = 60; 20 fish per replicate). All means between groups differed significantly from each sampling (*p* < 0.05). Letters only compare values within each column; means with common letters denote no significant differences (*p* < 0.05).

**Table 8:** Melanophore coverage (%) and melanin content (μg mm^−2^) in red seabream following experimental diet feeding over 30 and 60 days.

	Skin Area	Sampling	A25	A60	A80	A100	A0
Melanophore coverage	Dorsal	Day 30	1.2 ± 1.2	2.0 ± 0.6	2.4 ± 2.1	2.4 ± 1.3	2.1 ± 1.4
		Day 60	2.7 ± 1.3 ^b^	6.0 ± 4.0 ^a^	4.9 ± 1.8 ^a^	4.9 ± 2.2 ^a^	5.3 ± 1.9 ^a^
	Ventral	Day 30	0.01 ± 0.01	0.02 ± 0.03	0.005 ± 0.008	0.03 ± 0.04	0.003 ± 0.005
		Day 60	0.03 ± 0.07	0.02 ± 0.02	0.02 ± 0.02	0.01 ± 0.02	0.008 ± 0.01
Melanin	Dorsal	Day 60	23.6 ± 7.2	22.1 ± 4.8	19.6 ± 3.5	24.00 ± 5.9	26.4 ± 4.5

A0, A25, A60, A80 and A100 indicate the experimental feeds and the level of astaxanthin supplementation 0, 25, 60, 80 and 100 mg kg^−1^ of feed, respectively. Values are presented as mean ± SD (*n* = 15; *n* = 3 tanks). Mean values within the same row with no letters or with common letters denote no significant differences between the treatments (*p* > 0.05).

**Table 9:** Physicochemical indices of red sea bream’s plasma fed with experimental diets at day 60. Values are presented as mean ± SD. (*n* = 15; five fish per replicate).

	A25	A60	A80	A100	A0
Cholesterol (mmol L^−1^)	2.8 ± 0.95	2.98 ± 0.70	2.90 ± 0.88	2.93 ± 0.67	3.00 ± 0.92
Total proteins (mg dL^−1^)	0.51 ± 0.04 ^a^	0.48 ± 0.03 ^b^	0.46 ± 0.07 ^b^	0.56 ± 0.06 ^a^	0.43 ± 0.04 ^b^
Glucose (mmol L^−1^)	3.27 ± 1.34	2.92 ± 0.71	2.53 ± 0.56	3.14 ± 1.46	2.54 ± 0.29
Triglycerides (mmol L^−1^)	1.45 ± 0.84 ^a^	1.05 ± 0.35 ^a^	0.98 ± 0.66 ^a^	1.05 ± 0.42 ^a^	0.31 ± 0.10 ^b^
Cortisol (ng mL^−1^)	58.57 ± 51.2 ^ab^	31.92 ± 34.4 ^b^	69.9 ± 42.6 ^a^	69.9 ± 53.1 ^a^	43.31 ± 28.4 ^ab^
SOD (U mL^−1^)	1.74 ± 0.14	1.51 ± 0.22	1.57 ± 0.25	1.26 ± 0.11	1.41 ± 0.15
GPx (nmol min^−1^ mL^−1^)	83.82 ± 11.28	76.27 ± 22.44	82.36 ± 12.64	84.14 ± 22.70	98.12 ± 12.12

A0, A25, A60, A80 and A100 indicate the experimental feeds and the level of astaxanthin supplementation 0, 25, 60, 80 and 100 mg kg^−1^ of feed, respectively. Mean values within the same row with no letters or with common letters denote no significant differences between the treatments (*p* > 0.05).

## Data Availability

All data are available upon request from the authors.

## Supplementary Materials

The following supporting information can be downloaded at: https://www.mdpi.com/article/10.3390/ani16030499/s1, Indicative photos of fish fed the different feeding treatments from the second trial: Photo S1_A0: A0 treatment, Photo S2_A25: A25 treatment, Photo S3_A60: A60 treatment, Photo S4_A80: A80 treatment, Photo S5_A100: A100 treatment.

## Abbreviations

The following abbreviations are used in this manuscript:

OECD	Organization for Economic Co-operation and Development
LED	Light-emitting diode
VIT	Vitamin
