# Supplementary figures and images for: Effects of Different Sources and Dietary Inclusion Levels of Astaxanthin on Growth Performance, Skin Pigmentation, and Physiological Parameters of Red Sea Bream (Pagrus major) Juveniles
Source: Animals (Basel). 2026 Feb 5;16(3):499. doi: 10.3390/ani16030499 (PMC12896516; doi:10.3390/ani16030499)

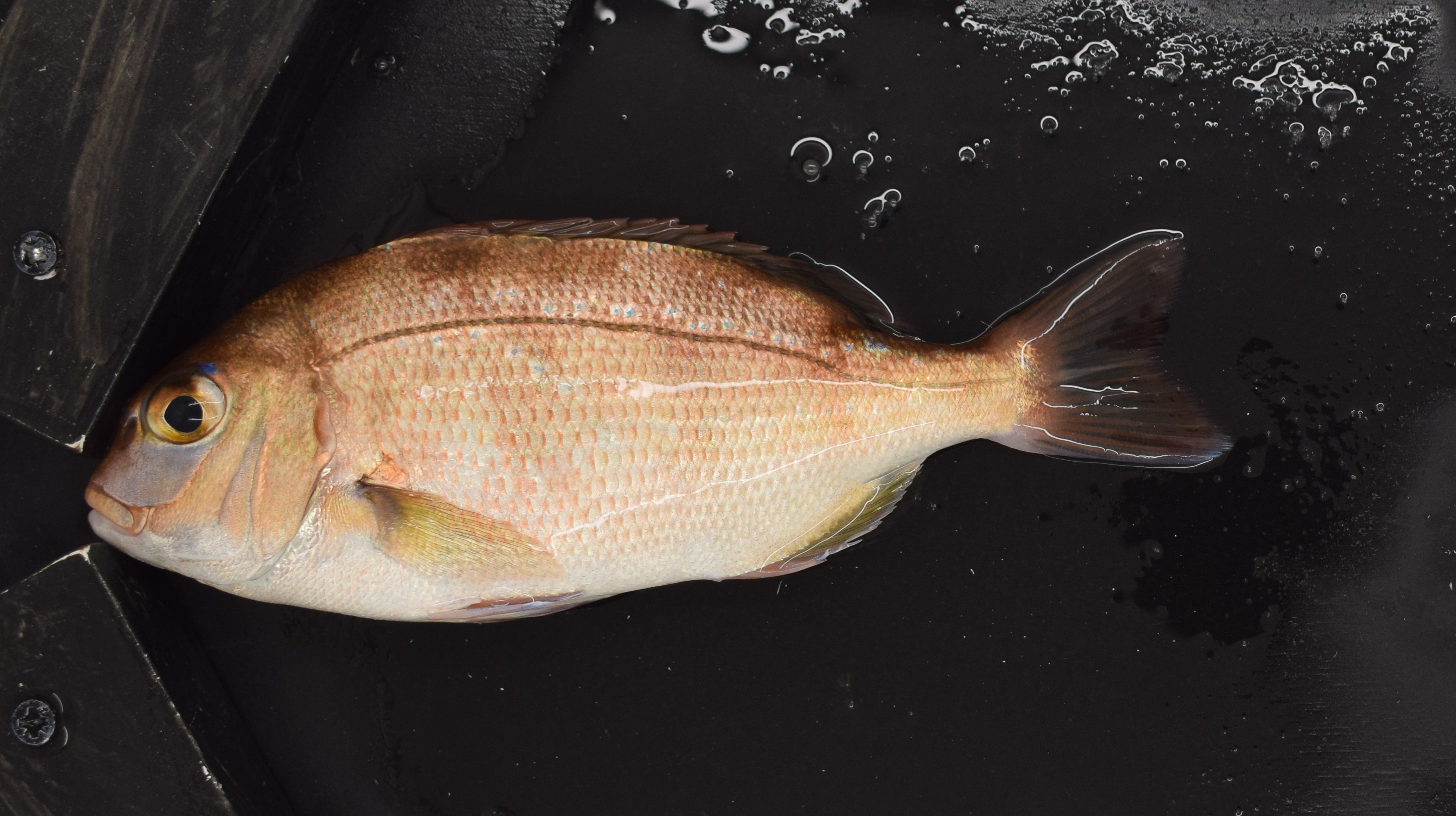

Supplement: Supplementary file 1 [file animals-16-00499-s001.zip › S5 A100.JPG]

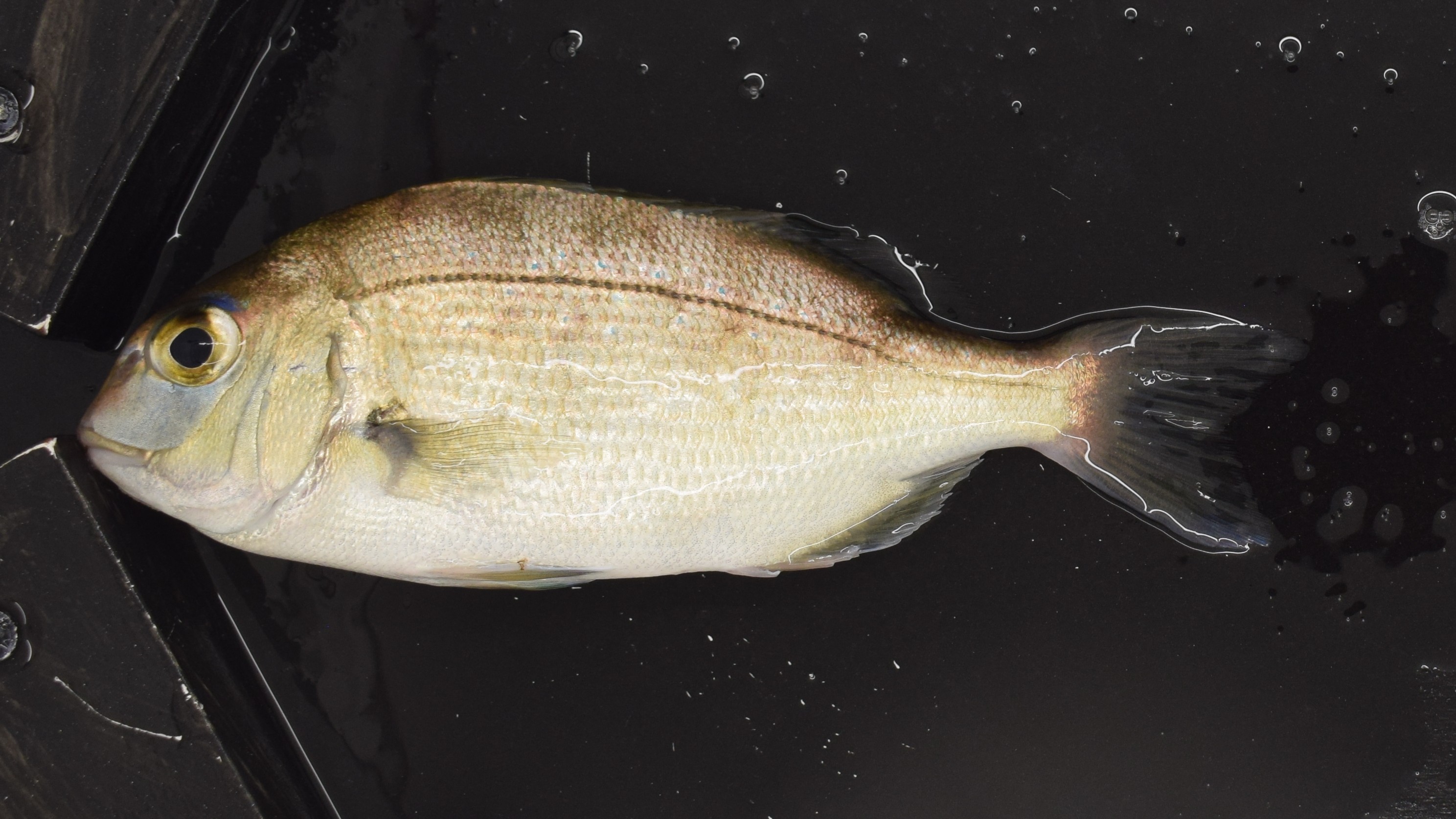

Supplement: Supplementary file 1 [file animals-16-00499-s001.zip › S1 A0.JPG]

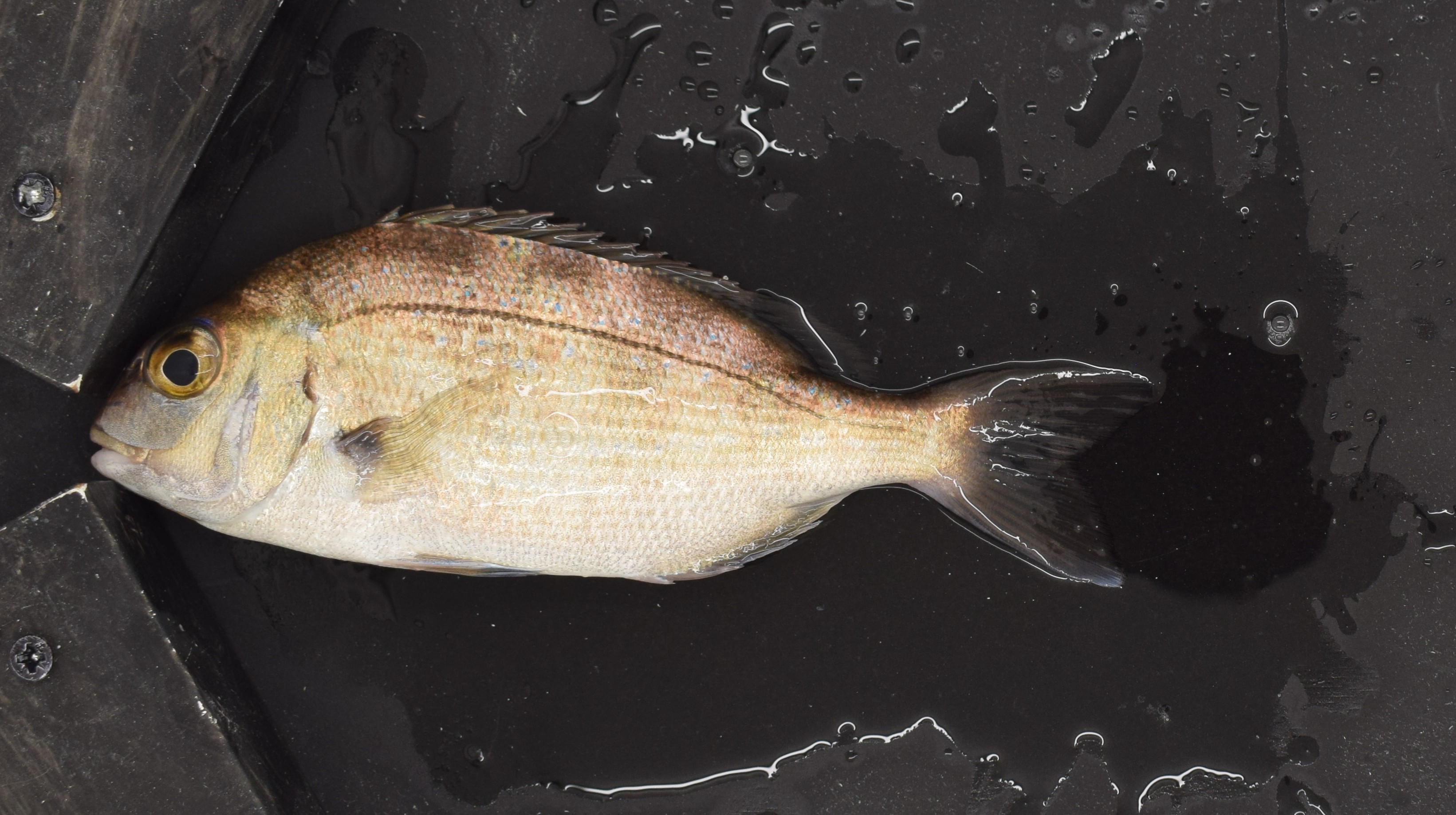

Supplement: Supplementary file 1 [file animals-16-00499-s001.zip › S2 A25.JPG]

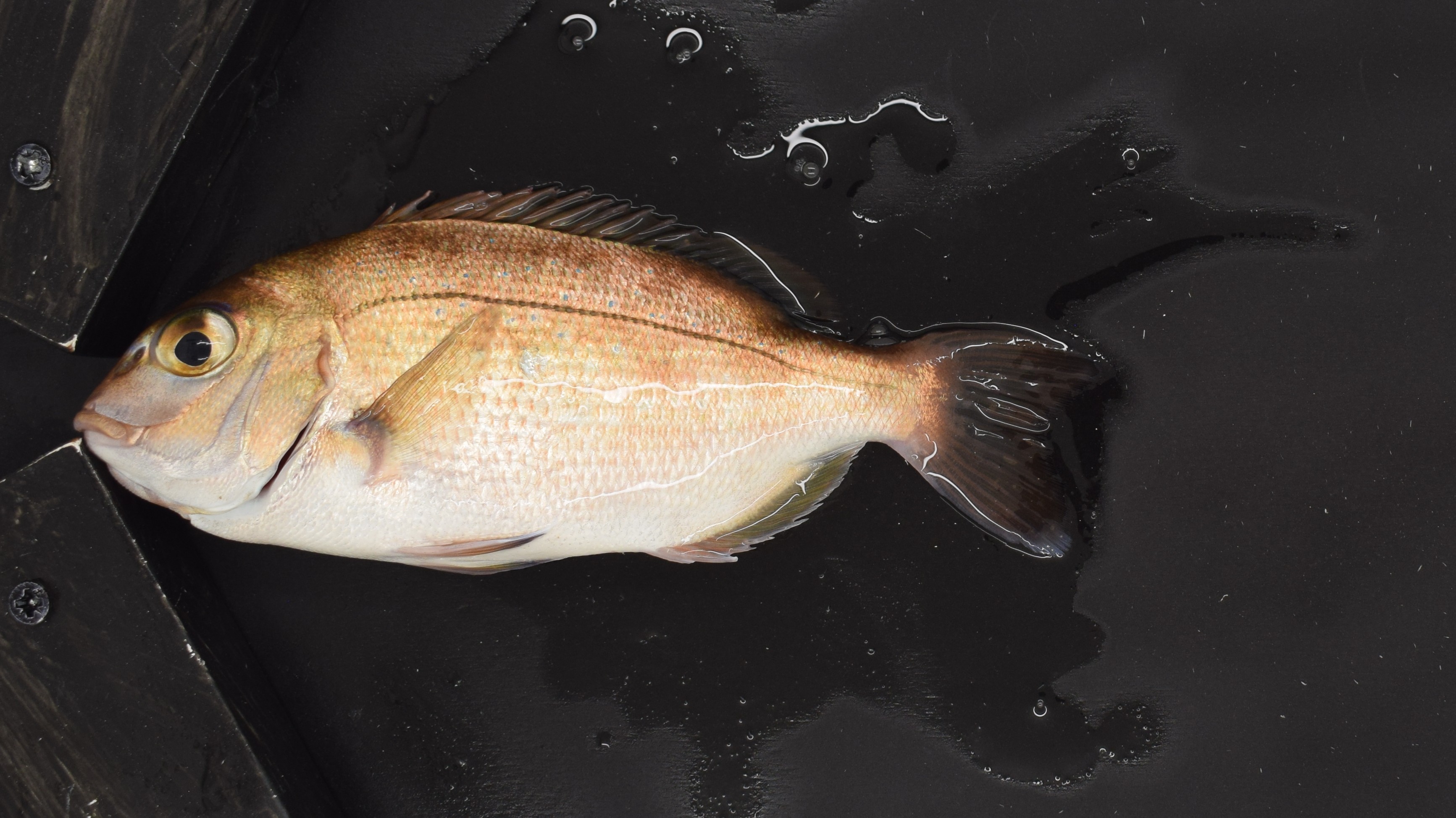

Supplement: Supplementary file 1 [file animals-16-00499-s001.zip › S3 A60.JPG]

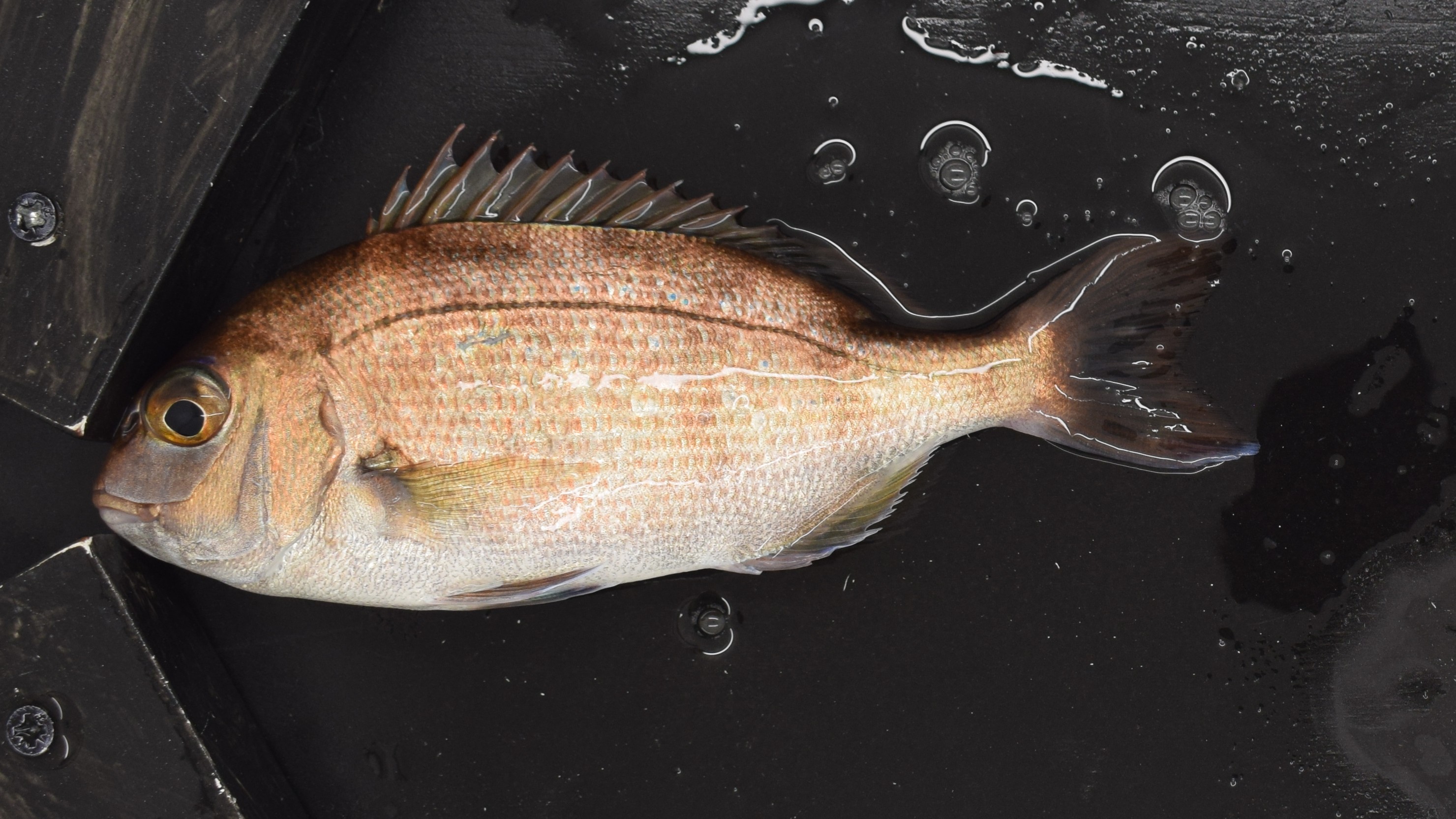

Supplement: Supplementary file 1 [file animals-16-00499-s001.zip › S4 A80.JPG]
